# Critical Review of Lead Pollution in Bangladesh

**DOI:** 10.5696/2156-9614-11.31.210902

**Published:** 2021-08-17

**Authors:** Ahmad Kamruzzaman Majumder, Abdullah Al Nayeem, Mahmuda Islam, Mohammed Mahadi Akter, William S. Carter

**Affiliations:** Center for Atmospheric Pollution Studies (CAPS), Department of Environmental Science, Stamford University Bangladesh

**Keywords:** lead, concentration, contamination, polluted, impacts, Bangladesh

## Abstract

**Background.:**

Lead (Pb) poses a severe threat to human health and the environment. Worldwide Pb production and consumption have significantly increased along with unplanned industrialization and urbanization, lead smelting, and lead-acid battery processing. The improper management of Pb-containing elements is responsible for Pb pollution. Lead's persistence in nature and bioaccumulation in the food chain can lead to adverse health impacts.

**Objectives.:**

The present study aims to describe Pb contaminated sites in Bangladesh and Pb concentration in the atmosphere, water, sediments, soil, vegetables, fish, and other foods in Bangladesh.

**Methods.:**

The present study searched a total of 128 peer-reviewed articles based on a predefined set of criteria (keywords, peer-reviewed journals, and indexing in Scopus, Science Direct, Web of Science, Springer, PubMed, Directory of Open Access Journals (DOAJ), and Bangladesh Journals Online (BanglaJOL) and exclusion criteria (predatory journal and absence of full text in English) and finally selected 63 articles (58 research articles and five (5) reports). The relevant findings on Pb exposure, sources, routes, diet, and impacts in Bangladesh were combined and presented.

**Results.:**

The reviewed studies identified 175 Pb contaminated sites through soil sample assessment in Bangladesh. The study determined Pb concentrations in air (0.09–376.58 μg/m^3^, mean 21.31 μg/m^3^), river water (0.0009–18.7 mg/l, mean 1.07 mg/l), river sediments (4.9–69.75 mg/kg, mean 32.08 mg/kg), fish (0.018–30.8 mg/kg, mean 5.01 mg/kg), soil (7.3–445 mg/kg, mean 90.34 mg/kg), vegetables (0.2–22.09 mg/kg, mean 4.33 mg/kg) and diet items (0.001–413.9 mg/kg, mean 43.22 mg/kg) of which 38.8%, 27.8%, 54.5%, 68.8%, 9.7% and 100% of samples, respectively, exceeded related World Health Organization (WHO), Food and Agriculture Organization (FAO), United States Environmental Protection Agency (USEPA) and Bangladesh Standard Testing Institution (BSTI) guidelines. The present study found that industrial soils are severely polluted with Pb (7.3–445 mg/kg) in Bangladesh. A high Pb concentration has been found in fish muscle and foods, including leafy and non-leafy vegetables collected from different places in Bangladesh.

**Conclusions.:**

Lead-contaminated foods can enter the human body through dietary intake and consequently lead to long-term adverse health effects. This study may help policymakers to formulate national policies with effective mitigation plans to combat the adverse health impacts of Pb in Bangladesh.

**Competing Interests.:**

The authors declare no competing financial interests.

## Introduction

The advancement of technology has led to the expansion of urbanization and industrialization, resulting in an upsurge of heavy metal pollution into the environment, especially in low- and middle-income countries (LMIC). Anthropogenic activities are a significant contributor to the amplification of heavy metal emissions into the earth's atmosphere, cultivated land, and water bodies.^1^

Rapid and unplanned urbanization and industrialization have led to toxic substances like arsenic (As), cadmium (Cd), chromium (Cr), copper (Cu), mercury (Hg), zinc (Zn), nickel (Ni), tin (Sn), and lead (Pb) being discharged to the surrounding environment.^2^ Planned urbanization also increases the risk of releasing toxic materials due to unregulated development activities.^3^ These metals contaminate the air, soil, and water through various pathways, including vehicle exhaust, fossil fuel combustion, suspended atmospheric particles, untreated municipal sewage, fertilizer and pesticides, municipal solid waste, and mining activities. Suspended air particles hold heavy metals, which eventually deposit onto the soil through the natural sedimentation and precipitation processes.^1^ Through various exposure routes such as diet, smoking, breathing, and drinking, toxic heavy metals can accumulate in various parts of the human body and cause severe health disorders.^3^

**Figure 1:**
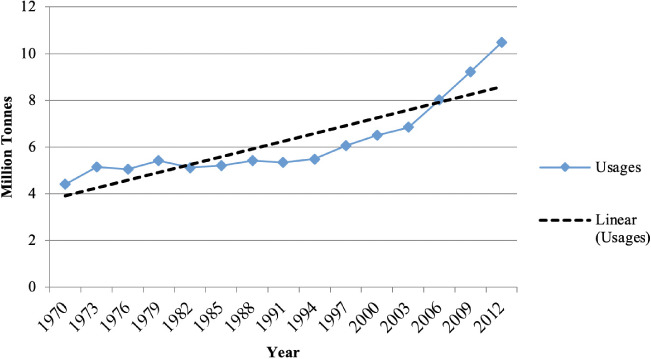
Global lead usage trend from 1970 to 2012^6^

Lead is an abundant non-biodegradable toxic heavy metal that is soft, corrosion resistant, highly malleable and ductile. As a result, Pb has been used in many industries. Lead has acute and chronic effects on human health and the environment.^4^ Due to the rapid growth of industrial activities, the high demand for lead-acid batteries (LAB) is now a concern in low- and middle-income countries.^3^ Since the early days of the industrial revolution, use of Pb has increased globally (*Figure 1*), and over the last decade, the exponential growth rate has become much more significant. In the 20^th^ century, Pb contamination has increased significantly due to the use of leaded gasoline in motor vehicles.^5^ The global usage of Pb in 2012 was 10.7 million tons^6^ (*Figure 1*). The demand for motor vehicles has led to increased LAB demand.^7^ This Pb is 100 percent recyclable, but the process is conducted chiefly through informal processes, especially in developing countries,^8^ resulting in soil contamination. The sector-based global annual consumption rate of Pb is shown in Table 1. Around 85% of Pb is used during the LAB manufacturing process. About 98.9% of used LAB (ULAB) were recycled in the United States in 2014, and around 99% in the European Union (EU) from 2010–2012.^6^

**Table 1:** Global Annual Consumption Rate of Lead (thousand tons)^6^

**Products**	**Consumption**	**%**
Batteries	9024	85.18
Cable sheathing	100	0.94
Rolled and extruded products	380	3.59
Shot/ammunition	150	1.42
Alloys	140	1.32
Pigments and other compounds	580	5.47
Miscellaneous	220	2.08

**Total**	**10 594**	**100**

Lead can have adverse environmental and health effects resulting from various anthropogenic activities. Table 2 summarizes the routes and activities that cause Pb contamination in the environment. Emissions from informal ULAB processing, automobiles that burn leaded gasoline, industrial dust, waste burning, open dumping, the paint industry, industrial sludge, mining activities, and low-grade fertilizer application are the leading causes of Pb contamination in air, soil, and water bodies.^9^ Soil can be contaminated with Pb through atmospheric deposition from various point sources like smelting and industrial processes.^3^ Several non-point sources such as fertilizers, sewage sludge, organic manure and compost are critical pathways of Pb contamination in vegetables and other crops.^10^

**Table 2:** Source Identification of Lead in the Environment

**Name of component**	**Major sources**	**Pathways**	**Reference**
Air	Automobile exhausts Industrial dust emission Building material damage Burning of solid waste Fumes from automobile exhaust Cement factory Fertilizer factory Fuel as anti-knock agent Tire wear and motor oils Smoke and dust emissions of coal and gas-fired power stations The laying of lead sheets by roofers as well as the use of paints and anti-rust agents Lead smelter industry Battery manufacturing industry Lead-based paint in buildings, bridges, and other structures Interior house dust	Atmospheric transmission and deposition	11–19

Water	Chemical mixed effluent from tannery industries Seepage wastes of riverside textile mills, dyeing Domestic solid waste Municipal wastes and pesticides Run off of wastes and atmospheric depositions Paint industry Industrial sludge Mining industry	Surface runoff and weathering	12,20–22

Soil	Agrochemicals (Pesticides, herbicides, Basudin, Rifit) Steel and iron industries Livestock manure, and unused metallic parts Dry cell batteries Used Pb acid batteries, Auto repair shops	Accumulation, sedimentation and deposition	2, 8,23,24

Others	Pb in cooking oil cans Pb in rice grinding and flour mills	-	8

**Abbreviations**
*LAB*: Lead acid batteries
*ULAB*: Used lead acid batteries
*WHO*: World Health Organization

Lead as an antiknock agent in petrol is one of the prime contributors to total atmospheric Pb pollution.^25^ Hence, people can be exposed to Pb through inhalation of Pb particles released from burning of Pb-containing material and or ingestion of dust, soil, diet, and drinking water that contains Pb. Lead intake can cause various health disorders, including kidney damage, DNA damage, change in blood composition, impaired hemoglobin synthesis, decreased red blood cell counts, and oxidative stress. Other adverse effects include impaired cognitive development in children, adverse effects on the development of the central nervous system, maternal death, mental retardation in children, and harmful effects on thyroid and growth hormones.^8^,^26^,^27^ Due to vulnerability to developmental effects, children are more greatly affected by Pb toxicity.^28^ Even low concentrations of Pb in human blood can cause adverse consequences.^29^ One study reported a Pb level in the blood of workers in battery manufacturing industries of around 47 μg/dL and 64 μg/dL in recycling plants in low- and middle-income countries, which exceeds the current United States standard of 5 μg/dL.^30^ Child laborers in low- and middle-income countries are at higher risk because of acute exposure to Pb as measured by blood Pb levels; levels exceeding 150 μg/dL may cause death.^31^,^32^ According to the Institute for Health Metrics and Evaluation (IHME) 2017,^33^ Pb exposure was responsible for 1.06 million deaths worldwide, with the highest Pb-related death rate in low- and middle-income countries. Lead not only affects the human body, but also has an adverse impact on soil microbial communities and the growth of plants.^23^ A summary of the effects of Pb poisoning on soil, plants, and humans is presented in Table 3.

**Table 3:** Effects of Lead Pollution on Environmental Components

**Source**	**Effects**	**Reference**
Soil	Inactivates enzymes of living cells Inhibits the uptake of essential nutrients by plant tissues from the soil Influences soil microorganisms by affecting their growth, morphology, and biochemical activities Affects soil enzyme activity Changes the composition of soil microbial communities Affects soil microbial respiration rate Inhibits microorganism reproduction Reduces synthesis and metabolism of microbial enzymes Decreases mineralization activity of microorganisms	17,34–36

Plants	Delays maturity and stunts growth of rice and wheat Reduces rice yield Retards benthos growth on the sea bottom Affects philological functions Retards nitrogen fixation, chlorosis, and metabolism Affects stoma function, photosynthesis activity and accumulation of other nutrient elements Damages root system	20,37–39

Human health	Impairs cognitive development of children Affects developing central nervous system Irreversibly decreases IQ Reduces energy levels Damages kidneys and DNA Alters gene expression Changes blood composition Impairs hemoglobin synthesis Impairs renal function, deafness, blindness, retardation Decreases libido, fatigue Neurobehavioral problems Changes the genetic code and causes rheumatoid arthritis Significantly decreases red blood cell counts, hemoglobin levels and hematocrit values	8,12,40–42

Bangladesh is a highly populated and polluted country.^25^ Along with rapid economic growth, it has experienced a dramatic shift in exposure to Pb over the past three decades. Bangladesh has a limited capacity for waste treatment and recycling facilities. Untreated wastes are discharged into nearby agricultural lands, rivers, roadside canals, or streams. Lead from these sources can persist in the soil and water bodies that humans can take up through the food chain. A previous study found that the deposition of Pb in soil, crops, water, air, and vegetables is higher in the vicinity of Bangladesh's industrial and urban areas.^3^ Since the 20th century, two and three-stroke engines have been major contributors to atmospheric Pb pollution in Bangladesh.^5^ There are 148 known informal recycling sites conducting ULAB and 97 LAB manufacturing sites in Bangladesh. This presents a threat to human health as people living near urban or industrial areas have higher blood Pb levels in their bodies.^8^ The present study aims to determine potential Pb contaminated sites in Bangladesh and Pb concentrations in different environmental spheres.

## Methods

A systematic literature search was conducted of research findings on Pb exposure from relevant sources such as peer-reviewed articles, textbooks, and reports of Pb contamination and poisoning in Bangladesh. The literature search focused on seven prioritized aspects of Pb pollution and pathways (atmosphere, water, sediment, fish, soil, vegetables, and foods) along with hotspots and impacts on living organisms. The search was performed through electronic databases using the following terms: “Pb in atmosphere,” “Pb pathway,” “Pb exposure,” “Pb in river water,” “trace metals in water,” “Pb in river sediment,” “Pb from industrial emission,” “heavy metal concentration in soil,” “Pb in food,” “Pb in vegetables,” “pathways and routes of Pb,” “Pb in the food chain,” “health effects of Pb,” “Pb effects on plants.”

One hundred and twenty-eight (128) related research papers were identified from accepted publication platforms around the world (Science Direct, PubMed, Institute for Scientific Information (ISI), Web of Science, Springer Link, National Center for Biotechnology Information (NCBI), Directory of Open Access Journals (DOAJ), and JSTOR); prominent national libraries (BanglaJOL, Department of Environment, Ministry of Health, International Center for Diarrheal Disease Research, Bangladesh (icddr,b), Bangladesh Atomic Energy Commission (BAEC), Bangladesh Standards & Testing Institution (BSTI)); and international organizations (Asian Development Bank, Clean Air Asia, Norwegian Institute for Air Research, School of Environmental Science Murdoch University, Australia, Stockholm Environment Institute (SEI), Sweden, World Health Organization (WHO), Pure Earth, International Lead Association (ILA), and the World Bank).

### Eligibility criteria

The following inclusion criteria were adopted: (i) peer-reviewed with mentioned database (ii) articles investigating Pb in the atmosphere, water, sediment, fish, soil, vegetables, and foods; (iii) full texts published in English; (iv) use of scientific analytical methods; (v) discussion and interpretation of the main findings; (vi) limitations of the study (e.g. process of data collection, lack of data, lack of ethics clearance, lack of coherence in data analysis) and (vii) conclusions and implications within the scope of the study design. There were no restrictions on the date of publication. Criteria for exclusion were: (i) publication in predatory journals (predatory journals defined as not indexed, rapid publication process, contact email address is non-professional and non-journal affiliated, peer review process and publication timelines too short etc.), and websites (ii) published papers with English abstracts but without full texts in English.

### Data processing

**Figure 2:**
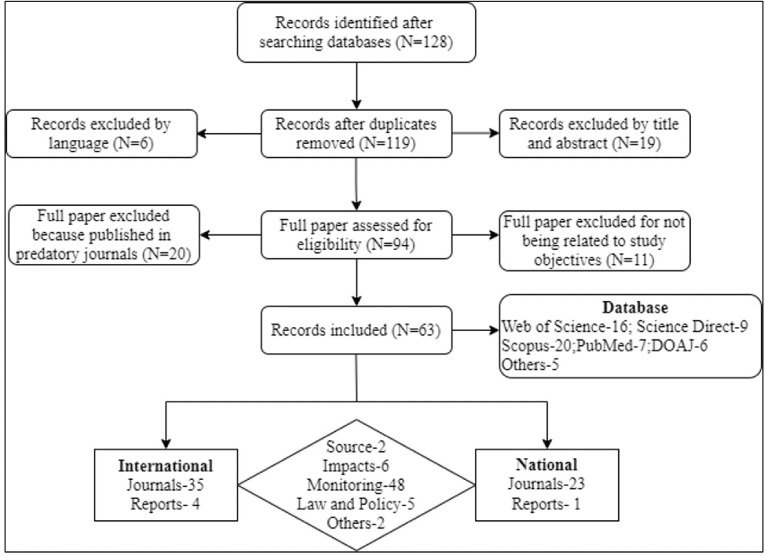
PRISMA flow diagram indicating Pb-related article collection, screening, inclusion and exclusion process

We followed two screening (abstract and full text) procedures on retrieved literature to determine article eligibility based on this study's objective. During the first screening, the title and abstract were reviewed based on Pb sources, route and impacts in Bangladesh. Next, the full text of those related abstracts was assessed to identify whether a study was fully or partially related to the study's objectives. Finally, 63 studies were selected for review. An overview of the literature selection procedure is shown in Figure 2. Lastly, findings were processed and analyzed following the cross-tabulation technique to list and compare Pb concentrations from various sources. For hotspot mapping and tabulation, ArcGIS 10.2.1 and Microsoft Excel 10 were used, respectively.

### Study characteristics

Table 4 presents the characteristics of the included papers. We considered 58 research articles from seven different media. In addition, five studies were examined for the standard concentration of various spheres. Two (2) of the 58 reviewed articles' key objectives was to classify the sources of heavy metal pollution, 48 articles described metal concentration, and six described health risks from Pb exposure. Lead concentrations in soil was the focus of 22 (32%) studies, and water and sediment results were reported in 12 papers (16%). Most of the first authors of these peer-reviewed articles were from Bangladesh, followed by the United States.

**Table 4:** Characteristics of Reviewed Papers

**Source**	**Number of papers**	**Literature theme (number of included papers)**	**First author region (number of included papers)**
Air	6 (8%)	Biomonitoring (4)^20^,^44^,^45^,^46^ Impact assessment (2)^25^,^32^	Bangladesh (5) United States (1)
Water	12 (16%)	Bimonitoring (11)^10^,^48^–^57^	Bangladesh (11)
Sediment	12 (16%)	Impact assessment (1)^22^ Biomonitoring-9^21^,^46^,^49^,^53^,^56^,^59^,^61^,^62^,^64^ Impact assessment (2)^22^,^63^ Others (1)^60^	Japan (1) Bangladesh (12)
Fish	7 (10%)	Source identification (1)^3^ Biomonitoring (5)^4^,^53^,^55^,^62^,^66^ Impact assessment (1)^67^	Bangladesh (7)
Soil	22 (30%)	Source identification (1)^81^ Biomonitoring (18)^2^,^5^,^11^,^12^,^15^,^20^,^48^,^57^,^70^–^77^,^79^,^80^ Impact assessment (2)^69^,^78^ Others (1)^7^	Bangladesh (20) Japan (1) China (1)
Vegetables	7 (10%)	Source identification (1)^3^ Biomonitoring (6)^15^,^83^–^17^	Bangladesh (6) Sweden (1)
Foodstuff	7 (10%)	Source identification (1)^3^ Biomonitoring (2)^74^,^89^ Impact assessment (4)^8^,^22^,^90^,^91^	Bangladesh (2) United States (3) Sweden (1) Japan (1)

Note: The sum of the studies is higher than 63 since some studies covered more than one sphere.

## Results

**Figure 3:**
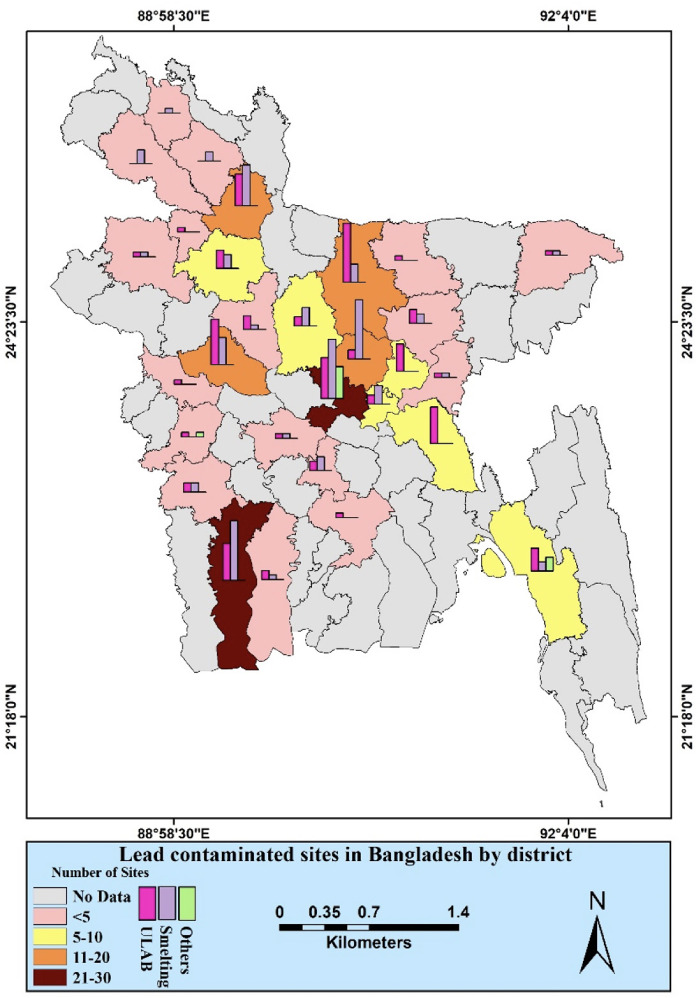
Map of district-wise Pb contaminated sites screened through the Toxic Sites Identification Program in Bangladesh between 2011 to 2018 (Adopted from McCartor, 2018)^43^

In collaboration with the Department of Geology of the University of Dhaka and the Department of Environment, Bangladesh, Pure Earth investigated Pb contaminated hotspot areas in Bangladesh. The investigators collected soil samples, following the Initial Site Screening (ISS) protocol provided by Pure Earth,^43^ and determined the hotspot zones based on the analyzed results. One hundred and seventy-five (175) of the assessed sites were found to be contaminated with Pb.^43^ Eighty-five (85) battery recycling/manufacturing/repairing and 84 Pb smelting industries were identified as sources of Pb pollution in Bangladesh. In addition, tannery and dye operations, heavy industry, chemical, and fertilizer manufacturing were identified as sources of Pb pollution in different districts of Bangladesh. Figure 3 shows the spatial distribution of Pb-contaminated hotspot areas in Bangladesh. Dhaka and Khulna districts were found to be the most polluted and have more smelting and ULAB industries than other districts.

### Lead in air

Table 5 shows Pb concentrations (μg/m^3^) in the ground-level atmosphere in Bangladesh. Woo, *et al.* (2018) recorded a Pb concentration of 376.58 μg/m^3^, the highest among the 18 studied areas, and around the battery manufacturing plant of Munshiganj Sadar, the Pb level of a nearby residential area was 1.22 μg/m^3^.^32^ Ahmed *et al.* (2012)^44^ determined Pb concentrations in places in Chittagong city and found that the New Market area of Chittagong city had the highest concentration of Pb (0.74 μg/m^3^). Rahman *et al.* (2013)^45^ showed that the air at various locations of Dhaka city had a range of 0.1–1 μg/m^3^. In summary, 9 out of 18 sites in Dhaka and its vicinity exceeded the Bangladesh National Ambient Air Quality Standard (BNAAQS) for Pb concentration in air. In Dhaka city, Tejgaon heavy industrial area had the highest concentration level in the present study. The mean concentration was 21.31 μg/m^3^, nearly 47 times higher than the standard set by BNAAQS. Used LAB and smelting industries, fertilizers, dye, cement, and paint industries, and vehicle exhausts were identified as primarily responsible for atmospheric Pb pollution in Bangladesh in these reviewed articles.^44^,^45^

**Table 5:** Lead Concentration (μg/m^3^) in Ground Level Atmosphere in Bangladesh

**District**	**Site description/Location**	**Concentration** (μg/m^3^)	**Reference**	**Arithmetic mean** (μg/m^3^)
Dhaka	Dhaka	0.29	25	21.31
Dhaka	Mukarram Hussain Khundkur Science Building, Department of Chemistry, University of Dhaka, Bangladesh	0.31	46	
Munshiganj	Residential area	1.22	32	
	Battery manufacturing plant	376.58		
Chittagong	New market	0.74	44	
	Bahaddarhat	0.42		
	Nasirabad	0.3		
	GEC Circle	0.16		
	Director’s office	0.55		
Dhaka	Farmgate	0.5	20	
Chittagong	Alfalah Housing Society, Khulshi	0.15		
Kustia	Daulotpur	0.26		
Noakhli	Karachi Bazar	0.09		
Faridpur	Faridpur Medical College	0.09		
Dhaka	KMHV, Dhaka University Campus	0.2	45	
	Tejgaon	1.0		
	Hazaribagh	0.1		
	Uttara model town	0.6		
Standard value for air (Annual mean)		0.5	47	

Abbreviations: KMHV, Kazi Motahar Hossain Bhaban

### Lead in surface water, sediment and fish

Three of the reviewed studies indicated that in Bangladesh, metal pollution, especially Pb, is increasing in river water and sediment.^22^,^48^,^49^ Table 6 presents the Pb concentrations in both river and lake water of Bangladesh. Most of the studies were in the industrialized areas on the river banks near Dhaka and Chittagong. Eighteen (18) water bodies were studied from 14 areas with the highest concentration found in the Dhaleshwari River near the Savar industrial area.^50^ Lead concentrations in most studied rivers exceeded the standard of irrigation water, especially rivers near Dhaka and Chittagong city and the rivers near to any urbanized or industrialized area. However, Mokaddes *et al.* (2013)^51^ reported that the water in the lakes of Dhaka city was acceptable. The arithmetic mean of the concentration of Pb in surface water was 1.07, 107-fold higher than the irrigation water standard and 21.4-fold higher than the inland water standard set by the Department of Environment (DoE).^47^ Untreated and partially treated effluents from industries were identified as the leading causes of Pb pollution in river water.^50^

**Table 6:** Lead Concentration in Surface Water in Bangladesh (mg/l)

**Sampling site**	**Water body**	**Concentration** (mg/l)	**Reference**	**Arithmetic mean** (mg/l)
Heavy industrial zone of Chittagong	Karnafuli River, Chittagong	0.14	52	1.07
Rajfulbaria area in Savar	Dhaleshwari River, Dhaka	18.7	50	
Tongi heavy industrial area	Turag River, Dhaka	0.015	22	
Balughat, Shawaryghat and Foridabad station	Buriganga River, Dhaka	0.06	53	
Belanagar and Drenerghat station	Old Brahmaputra River	0.11	48	
Industrial and municipal effluent discharge area	Khiru River, Mymensingh	0.02	54	
Ghorashal bridge, Katchpur bridge, Kerosene ghat	Shitalakhya, Dhaka	0.05	10	
Industrial discharge point	Rupsha River, Khulna	0.02	55	
Bogra district urbanized area	Karatoa River, Bogra	0.04	56	
Pasur River	Pasur River, Khulna	0.02	49	
Chilmari	Brahmaputra	0.04	57	
Dhaka metropolitan area	Dhanmondi Lake	0.0009	51	
	Ramna Lake	0.001		
	Crescent Lake	0.0009		
	Gulshan Lake	0.0009		
	Bonani Lake	0.001		
	Rampura Lake	0.005		
Standard for irrigation		.01	58	
Standard for inland water		.05	47	

The results of the analysis of Pb concentrations in river sediment are reported in Table 7. Ahmed *et al.* (2010)^53^ recorded the highest Pb concentration in Buriganga river sediment (69.75 mg/kg). In Turag, the concentration varied from 24–33.8 mg/kg. The next highest concentration (31.4 mg/kg) was found in the Bangshi River sediment near the Dhaka Export Processing Zone (Mohiuddin *et al.*, 2015).^21^ That study reported that in Dhaka city more than 5 thousand tons of solid wastes are produced every day from domestic sources, of which 63% were dumped in nearby rivers.^21^

**Table 7:** Lead Concentration in River Sediment (mg/kg) in Bangladesh

**Sampling location/City**	**River name**	**Concentration** (mg/kg)	**Ref.**	**Arithmetic mean** (mg/kg)
Fourteen (14) sediment samples were collected from different areas upstream of Dhaka	Buriganga	31.4	21	32.08
-	Turag	24	59	
Thirty-four (34) stations distributed uniformly all over the Ganges-Brahmaputra-Meghna basin	Padma	17	60	
-	Turag	33.8	61	
Heavy industrial zone of Chittagong	Karnafuli	4.9	62	
Tongi Industrial Area, Dhaka	Turag	26.3	22	
Balughat, Shawaryghat and Foridabad Station, Dhaka	Buriganga River	69.75	53	
Belanagar and Drenerghat station, Mymensingh	Old Brahmaputra	7.6	46	
Dhaka export processing zone	Bangshi	59.9	63	
Ten different stations from upstream to downstream of the river, Rajshahi	Karatoa	54	56	
Mongla, Khulna	Paira	49	64	
Patuakhali	Pasur	7.3	49	
Standard		31	65	

Seven research studies and monitoring programs on Pb accumulation in fish in Bangladesh were identified *(Table 8).* In these studies, 21 of 32 samples in various fish species from different rivers of Bangladesh exceeded the WHO food safety guidelines for Pb of 0.5 mg/kg. Fish samples collected from the Karwan Bazaar fish market had the highest concentration of Pb and the Pangus fish ranked highest with nearly 62 times more than the WHO standard.^66^ All the samples collected from the Buriganga and Karnafuli River also contained high concentrations of Pb with average concentrations 10 times higher than the standard. Fish were identified as a significant protein source in the human diet in two of the reviewed papers. These fish assimilate Pb through ingestion of suspended particulate matter from water, ingestion of food, and surface adsorption by both tissues and membranes.^67^,^68^

**Table 8:** Lead Concentration in Fish Species (mg/kg) in Bangladesh

**River**	**Local name/species**	**Genus and species**	**Concentration (mg/kg)**	**Reference**	**Arithmetic mean (mg/kg)**
Buriganga river	Chapila	*Gudusia chapra*	10.23	53	5.01
	Baila	*Glossogobius giuris*	9.91		
	Tatkeni	*Cirrhinus reba*	8.93		
	Taki	*Channa punctatus*	9.91		
	Tengra	*Mystus vittatus*	11.68		
	Batashi	*Pseudeutropius atherinoides*	9.18		
Korotoa River	Pangas	*Pangasius pangasius*	0.74	3	
Rupsha River	Chingri	*Asian tiger shrimp*	0.033	55	
	Tara baim	*Lesser spiny eel*	0.036		
	Gudusia chapra	*Indian river shad*	0.027		
	Tank goby	*Glossogobius giuris*	0.018		
	Trout barb	*Raiamas bola*	0.09		
Kawran Bazar fish market	Rui	*Labeo rohita*	15.33	67	
	Katla	*Catla catla*	15.86		
	Pangas	*Pangasius pangasius*	30.8		
Paira River	Koi	*Cyprinus rubrofuscus*	0.25	4	
	Shing	*Heteropneustes fossilis*	0.27		
	Kholisha	*Colisa fasciata*	0.18		
	Shoil	*Channa striata*	0.25		
	Foli	*Notopterus notopterus*	0.25		
	Hilsha	*Tenualosa ilisha*	0.51		
	Kachki	*Corica soborna*	0.37		
Karnafuly River	Poua	*-*	0.886	62	
	Chring	*-*	1.84		
	Tengra	*Mystus armatus*	2.86		
	Chapila	*Gudusia chapra*	7.7		
Buriganga River	Ticto barb	*Puntius ticto*	3.05	68	
	Pool barb	*Puntius sophore*	3.16		
	Chala punti	*Puntius chola*	2.32		
	Rohu	*Labeo rohita*	6.98		
	Bele	*Glossogobius giuris*	1.77		
*Standard*			0.5	66	

### Lead in soil samples, vegetables and diet

Twenty-two (22) studies reported soil samples contaminated with Pb in different districts of Bangladesh. The listed primary sources were atmospheric deposition from smelting, mining, and other industrial activities, and fertilizers, pesticides, sewage sludge, organic manures, and composts.^11^ Industrial sites, Pb smelting, and mining areas were found to be polluted, as seen in Table 9. The Pb concentration of soil exceeded the limit in only three areas: Narayanganj industrial area (445 mg/kg), Pb smelting area in Khulna (224.43 mg/kg), and mine-affected area in Dinajpur (433 mg/kg). The average concentrations for all the studied samples were within acceptable limits. Lead can remain in soil for thousands of years without any changes, although insect activity, plowing, adding compost, and other activities do lower Pb in soil over time.^36^

**Table 9:** Lead Concentration in Soil (mg/kg) in Bangladesh

**Soil category**	**District/City**	**Concentration** (mg/kg)	**Reference**	**Arithmetic mean** (mg/kg)
Road dust	Dhaka	67.6	11	90.36
Road dust from industrial area	Dhaka	36	69	
Soil from industrial area	Dhaka	98	20	
Soil from industrial area	Narayanganj	445	2	
Cultivated soil	Jashore	12.6	70	
	Tangail	64.8	71	
	Mymensingh	59.3	72	
	Bogra	9.6	5	
	Chittagong	7.3	73	
Dust from auto repair shop	Dhaka	54.4	12	
Crop soil	Gazipur	17.8	15	
Crop soil	Dhaka	97.5	74	
	Narayanganj	105.9		
	Norshindi	119		
	Gazipur	79		
	Chittagong	106.2		
	Sylhet	86.7		
Soil from industrial area	Tangail	12.1	75	
Soil from Pb smelting site	Khulna	224.43	7	
Soil from industrial area	Dhaka	21.9	76	
Commercial and residential areas	Pabna	21.29	77	
Mine affected farmland soil	Dinajpur	433	48	
Bank of Brahmaputra River	Kurigram	26.7	57	
Medical industry area	Barisal	26.55	78	
Garden Soil	Dhaka city	86.9	79	
	Chittagong	56.4		
	Rajshahi city	68.9		
	Khulna city	68.9		
Roadside soil from 20 locations	Dhaka city	45.6	80	
Road dust from Dhaka City	Dhaka	147.52	81	
Standards		200	82	

Eight (8) studies in Bangladesh reported high Pb concentrations in different types of vegetables *(Table 10).* Uddin *et al.* (2019)^83^ stated that Pb concentrations in vegetables were 1.6–13.1 mg/kg in the Satkhira district. High concentrations of Pb were found in vegetable samples collected from the surrounding area of Dhaka Export Processing Zone (DEPZ). All vegetable samples in this study exceeded the Food and Agriculture Organization (FAO)/WHO guideline and the average concentration was 433-fold higher than the standard. According to three of the reviewed articles, the main reason for the higher concentration was cultivating vegetables in Pb-contaminated soil.^83^,^84^,^85^

**Table 10:** Lead Concentration in Vegetables (mg/kg) in Bangladesh

**Sampling site**	**Common name**	**Concentration** (mg/kg)	**Reference**	**Arithmetic mean** (mg/kg)
Satkhira district	Cauliflower	3.4	83	4.33
	Tomato	11.3		
	Sweet gourd	13.1		
	Eggplant	1.6		
	Papaya	5.2		
Gazipur Industrial zone	Bottle gourd	2.66	15	
	Pumpkin	2.9		
Bogura district	Potato	1.5	3	
	Chili	1.8		
Surrounding Dhaka	Eggplant	11.97	84	
Export Processing Zone	Chili	13.81		
	Tomato	14.15		
	Lady's finger	15.72		
	Cabbage	22.09		
Industrial areas of Jhenaidah district	Tomato	0.41	85	
	Bean	0.53		
	Brinjal	0.54		
	Cabbage	0.26		
	Potato	0.57		
	Radish	0.49		
Leather industry area of Dhaka city	Spinach	11.48	86	
Around the Paira River, Patuakhali	Tomato	0.2	3	
	Potato	0.4		
	Green amaranth	1.2		
	Red amaranth	0.9		
	Brinjal	0.3		
	Bottle gourd	0.4		
	Chili	0.2		
	Carrot	0.5		
	Onion	0.4		
	Bean	1.0		
Industrial Area, Chittagong	Water spinach	0.73	87	
	Bottle gourd	1.16		
Standard		0.01	88	

One of the reviewed studies stated that diet, including cereals, vegetables, and seafood are the primary sources of Pb exposure in Bangladesh.^3^ Seven (7) studies in Bangladesh examined the concentration of Pb in different foods and dietary items (*Table 11*). Forsyth *et al.* (2019)^91^ reported a Pb concentration in turmeric powder that was up to 100 times greater than the Bangladesh Standard Testing Institution's (BSTI) limit of 2.5 mg/kg.^92^ Islam *et al.* (2015)^3^ found that almost all collected food samples exceeded BSTI limits.

**Table 11:** Lead Concentration in Food Stuff (mg/kg) in Bangladesh

**Food**	**Concentration** (mg/kg)	**Reference**	**Arithmetic mean** (mg/kg)
Rice (uncooked)	0.025	89	43.22
Turmeric powder	80	90	
Traditional medicine	0.001	91	
Egg albumen	0.03	74	
Egg yolk	0.045		
Eggshell	0.12		
Guava	1.2	3	
Wheat	4.8		
Maize	2.4		
Rice	1.9		
Cow milk	0.15		
Duck egg	0.10		
Mango	2.4		
Loose turmeric powder (market)	19	8	
Packaged turmeric powder (market)	4		
Loose turmeric powder (pigment-processed)	283.9		
Turmeric root (pigment-processed)	413.9		
Shrimp	0.82	22	

## Discussion

After the banning of two and three-stroke motor vehicles in 2002, the most significant Pb emission sources in Bangladesh are now industries that use substances containing Pb, mostly informal ULAB, and the smelting industry. Figure 3 shows that most of the contaminated sites are either ULAB or smelters. Heavy traffic congested areas and highly industrialized zones in Dhaka and Chittagong had atmospheric Pb levels exceeding the Bangladesh standard of 0.5 μg/m^3^.^47^ In Chittagong, the New Market and Director's office areas are two of the city's busiest areas, and road dust and vehicle exhaust were the primary sources of Pb pollution in Chittagong.^78^ Near a battery manufacturing plant in Munsiganj Sadar Upazila, including the surrounding residential areas, the concentration was higher than other locations.

Being a river-fed country, resources from rivers help the country's economic growth, but contaminated and polluted resources like water, sediment, and fish can harm humans through the food chain and bio-magnification process. The reviewed studies have shown that all rivers near to an industrial site, urbanized area, or port such as Dhaleswari, Buriganga, Karnafuli, Old Brahmaputra, and Shitalakhya, have Pb-polluted water except for the Turag River and Tongi Lake (joined to the Turag River). However, these two water bodies' contamination levels increased from 2012 to 2016.^22^,^61^ Lead concentrations in other rivers in this study, including the Khiru, Pashur, Brahmaputra, Rupsha, and Karatoa rivers, were found to be within the inland water standard and some exceeded the irrigation water standard.^58^ The water in the lakes of Dhaka metropolitan city had lower Pb levels due to regular maintenance and the absence of any industry adjacent to them.^51^ Sediments from some of the rivers have significant Pb levels. Surprisingly, two rivers' sediments, the Padma and Karnafuli, had Pb within the acceptable range even though the latter is adjacent to the urbanized port city of Chittagong, which has eight Pb production sites. The strong current and wave action of the Padma and Karnaphuli Rivers may reduce the potential for depositing sediment. Fish samples collected from the Buriganga, Karnafuli and Karwan Bazar contained high levels of Pb.^53^,^62^,^66^ The sediment of the Payra River in the Khulna area was found to be polluted with Pb, but the concentration of Pb in all the fish studied from this river was within the WHO food safety guidelines for Pb of 0.5 mg/kg, except Hilsha, the national fish of Bangladesh.^66^ The fish Pb levels captured from the Rupsha River in the Khulna area were also below the WHO food safety guidelines, whereas the water and sediment from this river were contaminated with Pb.^55^ It would seem the fish of coastal areas are safer to eat even though the water and sediment are polluted with Pb. The sources of the fish sold in the Karwan Bazar fish market were not stated, however it can be assumed that these fish are from anthropogenic sources cultured with Pb-contaminated food.^66^

In the present study, most of the surveyed soils were safe according to the United States Environmental Protection Agency (USEPA) standard.^82^ Nonetheless, all of them contained some contamination. Although atmospheric deposition is a major pathway of Pb concentration, the soil and dust of Dhaka city is seen to have Pb within the limit of the USEPA standard, but the vegetables grown on these soils had very high Pb levels exceeding the standard of FAO/WHO, 2011^88^ of .01 mg/kg. In Satkhira, the soil was not polluted with Pb but the Pb content in vegetables was 56–65-fold higher than the FAO/WHO food safety standard.^88^ This intrusion may be happening because of atmospheric deposition, irrigated water from nearby Pb-containing water bodies, Pb-loaded fertilizer, pesticide, organic manure, poultry feed, and compost.^3^ One of the reviewed studies indicated that the concentration of Pb in soil and vegetables decreased with increased distance from the roadside in Gazipur, Bangladesh indicating that traffic was the reason for increased concentrations of Pb in roadside soil and associated vegetables.^15^ The current study found that chicken egg, duck egg, and cow milk accumulated a significant amount of Pb due to accumulation from Pb-contaminated poultry feed.^1^ The elevated Pb levels in cereals and vegetables could be due to Pb smelting, emissions from vehicles and other industrial activities in the urban area and its vicinity.^3^ Elevated Pb concentrations in rice have become a global problem.^18^

Data were compiled into a frequency table (*Table 12*) to determine the most contaminated sector. Based on the percentage of contaminated samples, the most polluted sector was vegetables, where 100% of the studied samples were found to be contaminated with Pb followed by 68.8% of the fish species. Soil was found to be the least contaminated sector in Bangladesh. The authors did not find any direct relationship in Pb concentration between soil and associated vegetables or foodstuff. Since all the spheres of the environment are interrelated, there is a need for more research on Pb concentration in air, water, soil, and foodstuff grown in specific areas. Comparing the average concentration with the standards of each sector, the most polluted sector was found to be vegetables.

**Table 12:** Percentage of Samples Exceeding Standard

**Sphere**	**Total Sample**	**Standard Guideline**	**Exceeded Sample**	**Percentage of Exceeded Sample**
Atmosphere	18	ECR-0.5 μg/m^3^ 47	7	38.8
Surface water	18	ECR-0.05 mg/1^47^	5	27.8
River sediment	11	USEPA-31 mg/kg^65^	6	54.5
Fish species	32	WHO-0.5 mg/kg^66^	22	68.8
Soil	31	USEPA-200 mg/kg^82^	3	9.7
Vegetables	33	FAO/WHO-0.01 mg/kg^88^	33	100.0

Abbreviations: ECR, Environment conservation rules; FAO/WHO, Food and Agriculture Organization/World Health Organization; USEPA, United States Environmental Protection Agency

Several studies have reported that Bangladeshi populations are exposed to excessively high levels of Pb in their diet and through inhalation.^3^,^48^,^49^,^89^ This risk has been verified by blood lead level (BLL) in Bangladesh. Forsyth *et al.* (2019) found that 36 out of 45 pregnant women had a BLL greater than 5 μg/dL in three rural districts. Higher BLLs have been associated with the consumption of adulterated turmeric. Several studies found higher BLLs in the residents of urban areas like Dhaka and Narayanganj, industrialized areas, and rural agrarian regions (Munshigonj and Dinajpur).^8^,^9^,^32^ These studies tried to determine the significant pathways of Pb absorption in blood.

### Study limitations

Documents published in languages rather than English were excluded, and we focused on papers that were available online only, thus potentially excluding some useful sources. Another limitation of the study was the heterogeneity in the sample collection procedures in included papers. Despite these limitations, the present study can provide an overview of Pb pollution in Bangladesh.

## Conclusions

Lead contamination in the environment from various sources has become a major issue for the people of Bangladesh. This study demonstrates that Pb risk in Bangladesh is associated with ULAB, smelting, mining, and industrialization procedures. In the present study, the average Pb concentration was found to be 21.31 μg/m^3^ in the local air, 1.07 mg/l in river water, 32.41 mg/kg in river sediments, 5.01 mg/kg in fish, 90.36 mg/kg in soil, 4.33 mg/kg in vegetables, and 43.223 mg/kg in food items. Atmospheric Pb in Dhaka and Chittagong city was found to exceed the BNAAQS. Lead concentrations in residential areas adjacent to industrial zones may pose a serious health risk to the inhabitants, especially children. Since Bangladesh is a part of the great Ganga-Brahmaputra-Meghnaz basin, it drains a large volume of water through its narrow neck. That enables Pb to accumulate in the south of Bangladesh. Eventually, foodstuff like rice, wheat, maize, fruits, turmeric, fish, and vegetables may be contaminated with Pb, and in the present analysis, the most contaminated sector was found to be vegetables, (0.2–22.09 mg/kg) both in percentage and average concentration.

The food chain in Bangladesh is severely contaminated by Pb, which can contribute to fatal and chronic diseases through bio-concentration and bio-magnification. The present study found Pb in human blood in Bangladesh, which suggests adsorption through food stuff and the food chain. The adverse health effects of inhalation and ingestion of Pb are well known. Hence further research is needed to assess human exposure through consumption of potable water, fish, vegetables, and other foodstuff. Soil was the biggest potential source for Pb contamination in the reviewed papers. Additional studies are needed to determine the relationship between soil Pb level and Pb in associated vegetables and other agricultural products. Since the toxicity of Pb depends on its chemical state, a study could be initiated to determine the solubility of inorganic lead in contaminated locations and determine if there are any residual amounts of organic lead (tetraethyl lead). The appropriate authority should take mitigation measures to stop the unauthorized operation of Pb smelters and ULAB. Institutional frameworks and national policies are required to combat the adverse health impacts of Pb pollution in Bangladesh.
